# Supplementary material for: Development and Characterization of an Edible Zein/Shellac Composite Film Loaded with Curcumin
Source: Foods. 2023 Apr 7;12(8):1577. doi: 10.3390/foods12081577 (PMC10137614; doi:10.3390/foods12081577)
Supplement: Supplementary file 1 [file foods-12-01577-s001.zip › foods-2291187 - Supplementary Materials.pdf]

Supporting Information

Development and characterization of an edible zein/shellac composite film loaded with curcumin

Tao Han[1], Wenxue Chen[1], Qiuping Zhong[1], Weijun Chen[1], Yaping Xu[1], Jiawu Wu[1], Haiming Chen[1,2,*]

[1] *HNU-HSF/LWL Collaborative Innovation Laboratory, School of Food Science and Engineering, Hainan University, 58 Renmin Road, Haikou, 570228, PR China*

[2]*Maritime Academy, Hainan Vocational University of Science and Technology, 18 Qiongshan Road, Haikou, 571126, PR China*

***Corresponding Authors:**

Haiming Chen E-mail addresses: hmchen168@126.com (H. Chen)

Hainan University-HSF/LWL Collaborative Innovation Laboratory, School of Food Science and Engineering, Hainan University, 58 Renmin Road, Haikou 570228, PR China.

Table S1. The coefficient estimates of single factor test.

Single factor	Level	WVP ($g·mm·m^{-2}·h^{-1}·kPa^{-1}$)	WS (%)	TS (MPa)	EB (%)	PIF
Zein / Shellac (ratio)	3.5:0.5	1.718±0.340^{a}	16.828±2.390^{a}	0.521±0.096^{b}	0.212±0.035^{b}	0.018
	3:1	1.289±0.285^{b}	17.455±1.498^{a}	0.518±0.211^{b}	0.222±0.010^{b}	0.092
	2.5:1.5	1.020±0.104^{c}	10.580±1.019^{b}	0.610±0.211^{b}	0.524±0.155^{b}	0.292
	2:2	0.432±0.086^{d}	9.889±1.989^{b}	0.926±0.303^{a}	1.254±0.099^{b}	0.682
	1.5:2.5	0.403±0.043^{d}	9.682±0.660^{b}	1.004±0.088^{a}	3.337±2.156^{ab}	0.803
	1:3	0.138±0.008^{e}	7.183±1.220^{c}	0.505±0.082^{b}	8.233±1.182^{a}	0.600
Ethanol concentration (%, v/v)	75	0.773±0.063^{b}	19.929±3.316^{a}	0.911±0.078^{c}	0.620±0.147^{b}	0.374
	80	0.812±0.058^{b}	19.646±1.731^{a}	0.965±0.086^{bc}	0.996±0.356^{b}	0.380
	85	0.853±0.175^{b}	18.110±1.467^{a}	1.118±0.048^{b}	1.194±0.125^{b}	0.447
	90	0.206±0.024^{c}	16.962±2.771^{ab}	0.864±0.182^{c}	1.302±0.228^{b}	0.686
	95	0.239±0.034^{c}	20.024±4.028^{a}	1.476±0.163^{a}	1.441±0.315^{b}	0.594
	100	1.749±0.180^{a}	13.118±1.545^{b}	0.257±0.039^{d}	19.375±2.602^{a}	0.400
Plasticizer type	TBC	0.567±0.038^{c}	13.128±1.058^{c}	0.698±0.038^{a}	0.876±0.144^{a}	0.976
	PEG400	1.879±0.073^{a}	14.018±0.825^{c}	0.785±0.050^{a}	0.750±0.004^{ab}	0.443
	Glycerin	1.029±0.018^{b}	17.670±1.348^{b}	0.430±0.189^{b}	0.496±0.002^{c}	0.502
	Tween20	0.575±0.027^{c}	24.274±1.386^{a}	0.665±0.034^{a}	0.621±0.149^{bc}	0.596
TBC added amount (%, w/w)	5	0.549±0.094^{ab}	10.952±0.596^{d}	0.943±0.137^{c}	0.803±0.123^{c}	0.491
	10	0.627±0.043^{a}	11.661±0.333^{cd}	1.026±0.080^{bc}	0.938±0.124^{b}	0.372
	20	0.508±0.031^{b}	12.619±1.222^{bcd}	1.207±0.085^{b}	1.121±0.136^{a}	0.514
	30	0.403±0.025^{c}	15.628±0.908^{a}	0.850±0.067^{c}	0.996±0.007^{ab}	0.405
	40	0.249±0.013^{d}	12.999±1.046^{bc}	1.576±0.316^{a}	0.738±0.008^{c}	0.810
	50	0.383±0.111^{c}	14.202±1.782^{ab}	0.568±0.129^{d}	0.473±0.015^{d}	0.413
OA added amount (%, w/w)	10	0.742±0.051^{a}	12.115±0.901^{c}	0.982±0.132^{b}	0.879±0.144^{c}	0.326
	15	0.699±0.188^{a}	16.170±1.606^{b}	1.248±0.155^{a}	1.240±0.184^{b}	0.339
	20	0.475±0.021^{bc}	15.217±2.463^{b}	1.087±0.327^{b}	0.937±0.124^{c}	0.619
	25	0.322±0.039^{c}	10.640±1.062^{c}	1.073±0.154^{b}	0.867±0.140^{c}	0.884
	30	0.479±0.047^{bc}	15.980±0.452^{b}	1.011±0.092^{b}	0.749±0.002^{c}	0.554
	35	0.621±0.154^{ab}	20.979±2.030^{a}	0.695±0.063^{c}	1.497±0.005^{a}	0.319

Note: Numbers are mean ± standard deviation (n = 4), different superscript letters within a column indicate significant differences ($p < 0.05$). PIF: The performance index of the film.

The effect of the Z/S mass ratio on the properties of the ZS film is shown in Table 1S. The WVP values of the ZS films decreased with increasing shellac content, indicating that the water vapor barrier performance of the film was improved. The trend

for changes in the WS was similar to that for the WVP. As the proportion of shellac was increased, the WS of the composite film decreased. When the mass ratio of Z/S was 1.5:2.5, the TS value of the ZS film was the highest, which suggested that the interactions between zein and shellac molecules resulted in formation of numerous hydrogen bonds. The EB value of the ZS film increased significantly ($p < 0.05$) as the proportion of shellac was increased, which was caused by the high viscosity of the shellac (Saberi, et al., 2017). In the present study, the optimal ZS mass ratio was 1.5:2.5, as obtained by a comprehensive evaluation method (Statistical mathematics).

Ethanol is a good solvent for dissolving zein and shellac (Pena-Serna & Lopes, 2013; Yuan, et al., 2021), and the properties and structures of the composite films formed by both were also affected by the concentration of ethanol. As shown in Table 1S, the WVP value of the ZS film was the lowest when the ethanol concentration was 90%. The TS value of the ZS film was the highest when the ethanol concentration was 95%. The EB value of the ZS film was the highest when the ethanol concentration was 100%. Changes in the ethanol concentration had no obvious effect on the WS values of the ZS films. These results indicated that the higher the ethanol concentration was, the greater the solubility of shellac, while the solubility of zein decreased obviously when the ethanol concentration was increased to 100%, which made the ZS film formed at 100% ethanol concentration have strong ductility and poor tensile strength. The comprehensive evaluation showed that the best performance was obtained when the ethanol concentration was 90%, giving a PIF value of 0.686.

It has been reported that the use of appropriate amounts of plasticizers reduced the brittleness values of biopolymers and increased the fluidities of biopolymer chains (Otoni, et al., 2017). In this study, four different types of plasticizers, TBC (T), PEG-400 (P), glycerol (G), and Tween-20 (W), were used to analyze the effects of the plasticizer on the physical properties of the composite films. Table 1S shows that the ZS-T film had the lowest WVP and WS values due to the hydrophobicity of TBC (Andreuccetti, et al., 2009). The ZS-P film had the highest WVP and TS values attributed to the hydrophilicity of PEG-400， indicating that PEG-400 could improve the mechanical properties of the composite films (Soradech, et al., 2013). The comprehensive evaluation showed that the films prepared with TBC exhibited the best performance, and the corresponding PIF was 0.976.

The results in Table 1S indicated that the WVP, WS, TS and EB values of the ZS films all changed significantly ($p < 0.05$) with increasing TBC content, and the ZS-T40 film had good water vapor barrier and high tensile strength. Moreover, with the increase of TBC content, the TS and EB values of the ZS film first increased and then decreased, the EB value of the film was the largest when the TBC content was 20%, and the TS value of the film was the largest when the TBC content was 40%. These results indicated that the addition of TBC improved the ductility of the ZS film, but the effect on TS was small. Therefore, the adding the appropriate amount of TBC plasticizer significantly improved the mechanical properties of the composite film. Based on the PIF results, the optimal proportion of TBC was 40%, giving a PIF value of 0.810.

Oleic acid is a general lipid compound with strong water resistance (Ren, et al., 2019). Adding it to the film-forming solution improved the water resistance and flexibility of the film (Budi Santosa & Padua, 1999; Wang & Padua, 2005).

The effects of OA addition on the properties of the films are displayed in Table 1S. The lowest WVP and WS values for ZS films were obtained when 25% OA was added, and the film had the best water barrier properties. This result was consistent with the findings of Padua et al. (Gennadios, 2002), who reported that composite films prepared from zein and oleic acid showed significantly reduced WVP and WS values. In addition, the composite films exhibited better TS and EB values when OA was added at 15%, indicating that the addition of a small amount of OA significantly improved the mechanical properties of the composite films ($p < 0.05$). When the amount of OA added was 25%, the PIF value was 0.884, and the comprehensive performance was better than those of the other films.

Table S2. Factors and levels of orthogonal test.

Level	A<br>(Z/S ratio, g/g)	B<br>(Ethanol %, v/v)	C<br>(TBC %, w/w)	D<br>(OA %, w/w)
1	1:3	85	30	20
2	1.5:2.5	90	40	25
3	2:2	95	50	30

Table S3. The result of orthogonal experiment.

Formula	A	B	C	D	WVP (g·mm·m^{-2}· h^{-1}·kPa^{-1})	WS (%)	TS (MPa)	EB (%)	PIF
1	1	1	1	1	0.46±0.02^{c}	17.30±0.38^{a}	0.45±0.04^{g}	0.411±0.016^{f}	0.37
2	1	2	2	3	0.29±0.03^{f}	12.58±0.27^{c}	1.22±0.03^{a}	0.558±0.026^{d}	0.84
3	1	3	3	2	0.47±0.02^{c}	11.33±0.21s^{d}	0.86±0.02^{d}	0.741±0.002^{b}	0.70
4	2	1	2	2	0.67±0.02^{b}	14.25±0.27^{b}	0.52±0.01^{f}	0.290±0.017^{g}	0.28
5	2	2	3	1	0.68±0.02^{b}	11.55±0.17^{d}	0.69±0.03^{e}	0.740±0.010^{b}	0.47
6	2	3	1	3	0.46±0.03^{c}	9.81±0.08^{e}	1.13±0.05^{b}	0.494±0.005^{e}	0.76
7	3	1	3	3	0.81±0.01^{a}	17.76±0.70^{a}	0.46±0.02^{g}	0.429±0.024^{f}	0.02
8	3	2	1	2	0.34±0.02^{e}	11.07±0.35^{d}	1.06±0.08^{c}	0.931±0.032^{a}	0.88
9	3	3	2	1	0.41±0.02^{d}	12.92±0.38^{c}	1.08±0.03^{bc}	0.670±0.021^{c}	0.70
K1	1.91	0.67	2.02	1.55					
K2	1.51	2.19	1.82	1.85					
K3	1.61	2.16	1.19	1.62					
k1	0.64	0.22	0.67	0.52					
k2	0.50	0.73	0.61	0.62					
k3	0.54	0.72	0.40	0.54					
Range	0.13	0.50	0.28	0.10					
Influence		B>C>A>D							
Optimal level	A1	B2	C1	D2					

Each value in the table is the average and standard deviation of the repeat experiment for three times. Different letters mean significant difference ($p \leq 0.05$). PIF: The performance index of the film.

Based on the results obtained from the single-factor tests, the orthogonal experiment was designed. In the orthogonal experiment, four factors, including the Z/S weight ratio, ethanol concentration, and amounts of TBC and OA added, were selected. A four-factor three-level orthogonal test was carried out. The detailed experimental parameters are shown in Table 2S and Table 3S. The film properties of the nine groups varied significantly. Among them, formula 2 had the best water barrier properties and tensile strength, and formula 8 showed better performance for elongation at break. However, for water solubility, formula 6 was better.

According to the analytical results, the influence degrees of the four factors on film performance decreased in the order: ethanol concentration > TBC addition > Z/S mass ratio > OA addition. Moreover, it can be seen that the PIF values of the films ranged from 0.02 to 0.88, and the optimum formula for the ZS film was the fifth group. Therefore, the optimum formulation of the composite film was as follows: Z/S mass ratio 2:2, ethanol concentration 90% (v/v), TBC 30% (w/w), and OA 25% (w/w).

References

87 Andreuccetti, C., Carvalho, R. A., & Grosso, C. R. F. (2009). Effect of hydrophobic plasticizers on functional properties of gelatin-based films. *Food Research International, 42*(8), 1113-1121.

88 Budi Santosa, F. X., & Padua, G. W. (1999). Tensile properties and water absorption of zein sheets plasticized with oleic and linoleic acids. *Journal of agricultural and food chemistry, 47*(5), 2070-2074.

89 Gennadios, A. (2002). *Protein-based films and coatings*: CRC press.

90 Otoni, C. G., et al. (2017). Recent Advances on Edible Films Based on Fruits and Vegetables-A Review. *Comprehensive Reviews in Food Science and Food Safety, 16*(5), 1151-1169.

91 Pena-Serna, C., & Lopes, J. F. (2013). Influence of ethanol and glycerol concentration over functional and structural properties of zein-oleic acid films. *Materials Chemistry and Physics, 142*(2-3), 580-585.

92 Ren, X. F., et al. (2019). Effects of frequency ultrasound on the properties of zein-chitosan complex coacervation for resveratrol encapsulation. *Food Chemistry, 279*, 223-230.

93 Saberi, B., et al. (2017). Development of biocomposite films incorporated with different amounts of shellac, emulsifier, and surfactant. *Food Hydrocolloids, 72*, 174-184.

94 Soradech, S., Limatvapirat, S., & Luangtana-Anan, M. (2013). Stability enhancement of shellac by formation of composite film: Effect of gelatin and plasticizers. *Journal of Food Engineering, 116*(2), 572-580.

95 Wang, Q., & Padua, G. W. (2005). Properties of zein films coated with drying oils. *Journal of agricultural and food chemistry, 53*(9), 3444-3448.

96 Yuan, Y., et al. (2021). Shellac: A promising natural polymer in the food industry. *Trends in Food Science & Technology, 109*, 139-153.
